# Implication of *NOTCH1* gene in susceptibility to anxiety and depression among sexual abuse victims

**DOI:** 10.1038/tp.2016.248

**Published:** 2016-12-13

**Authors:** I M Steine, T Zayats, C Stansberg, S Pallesen, J Mrdalj, B Håvik, J Soulé, J Haavik, A M Milde, S Skrede, R Murison, J Krystal, J Grønli

**Affiliations:** 1 Department of Psychology, University of California, Berkeley, Berkeley, CA, USA; 2 K.G. Jebsen Centre for Neuropsychiatric Disorders, Department of Biomedicine, University of Bergen, Bergen, Norway; 3 Dr. Einar Martens Research Group for Biological Psychiatry, Center for Medical Genetics and Molecular Medicine, Haukeland University Hospital, Bergen, Norway; 4 Genomics Core Facility, Department of Clinical Science, University of Bergen, Bergen, Norway; 5 Department of Psychosocial Science, University of Bergen, Bergen, Norway; 6 Norwegian Competence Center of Sleep Disorders, Haukeland University Hospital, Bergen, Norway; 7 Department of Biological and Medical Psychology, University of Bergen, Bergen, Norway; 8 The Norwegian Centre for Mental Disorders Research (NORMENT) and the K.G. Jebsen Centre for Psychosis Research, Department of Clinical Science, Haukeland University Hospital, Bergen, Norway; 9 Department of Biology, University of Bergen, Bergen, Norway; 10 Division of Psychiatry, Haukeland University Hospital, Bergen, Norway; 11 Regional Centre for Child and Youth Mental Health and Child Welfare, Bergen, Norway; 12 Clinical Neuroscience Division, VA National Center for PTSD, West Haven, CT, USA; 13 Department of Psychiatry, Yale University School of Medicine, New Haven, CT, USA; 14 Elson S. Floyd College of Medicine, Washington State University, Spokane, WA, USA

## Abstract

Sexual abuse contributes to the development of multiple forms of psychopathology, including anxiety and depression, but the extent to which genetics contributes to these disorders among sexual abuse victims remains unclear. In this translational study, we first examined gene expression in the brains of rodents exposed to different early-life conditions (long, brief or no maternal separation). Hypothesizing that genes revealing changes in expression may have relevance for psychiatric symptoms later in life, we examined possible association of those genes with symptoms of anxiety and depression in a human sample of sexual abuse victims. Changes in rodent brain gene expression were evaluated by means of correspondence and significance analyses of microarrays by comparing brains of rodents exposed to different early-life conditions. Tag single-nucleotide polymorphisms (SNPs) of resulting candidate genes were genotyped and tested for their association with symptoms of anxiety and depression (Hospital Anxiety and Depression Scale) in a sample of 361 sexual abuse victims, using multinomial logistic regression. False discovery rate was applied to account for multiple testing in the genetic association study, with *q*-value of 0.05 accepted as significant. We identified four genes showing differential expression among animals subjected to different early-life conditions as well as having potential relevance to neural development or disorders: Notch1, Gabrr1, Plk5 and Zfp644. In the human sample, significant associations were observed for two *NOTCH1* tag SNPs: rs11145770 (OR=2.21, *q*=0.043) and rs3013302 (OR=2.15, *q*=0.043). Our overall findings provide preliminary evidence that *NOTCH1* may be implicated in the susceptibility to anxiety and depression among sexual abuse victims. The study also underscores the potential importance of animal models for future studies on the health consequences of early-life stress and the mechanisms underlying increased risk for psychiatric disorders.

## Introduction

Sexual abuse is a potentially devastating event happening at epidemic rates worldwide. Between 8 and 31% women and 3 and 17% men have experienced sexual abuse during childhood^1^ or adulthood.^2^ The rates reported in Norway are comparable.^3^ The stressful nature of sexual abuse is evidenced by a large body of literature linking sexual abuse to a life-long increased risk to a wide range of somatic and mental health problems, independent of the victims' age at the time of the abuse.^4, 5, 6, 7, 8^ Furthermore, adults who were sexually abused during childhood display structural and functional alterations of brain areas and neuroendocrine systems involved in stress response regulation,^9, 10, 11, 12, 13^ as well as epigenetic modifications of genes involved in stress regulation.^14, 15^ Such changes provide potential neurobiological mechanisms underlying the association between childhood sexual abuse, or other types of early-life stress, and the development of disorders later in life.^9, 16, 17, 18, 19, 20^ The understanding of the neurobiology of early-life stress is still rather limited, however. Stress-induced sustained elevations in glucocorticoid levels may contribute to both structural and functional changes in the brain.^21, 22^ Such changes may include pathological alterations in secretion patterns of the endocrine system, including melatonin and peptide substances of the pineal gland^23^ and glucocorticoids of the hypothalamic–pituitary–adrenal system.^22^ Both the pineal gland and the hypothalamic–pituitary–adrenal system contribute to the general defense responses, with glucocorticoids postulated to modulate the activity of the pineal gland.^24, 25^ Moreover, the antistress properties of the pineal gland have been reported to be mediated by the functional state of the hippocampus,^26^ a brain structure especially sensitive to glucocorticoids. This brain region has a key role in memory consolidation, cognition and mood. It is also among the few brain areas capable of producing new neurons throughout life.^27^ Sustained elevations in glucocorticoid levels have been reported to suppress hippocampal neurogenesis,^28^ reduce the number of dendritic spines and produce dendritic atrophy.^29^ These changes may contribute to the behavioral impact of early-life stress.^22^ Correspondingly, clinical neuroimaging studies have shown hippocampal atrophy in adolescents and adults exposed to childhood sexual abuse,^11, 12, 13^ suggesting the possible involvement of the hippocampus in the pathophysiology triggered by this adversity.^22^ Nonetheless, the cellular mechanisms behind behavioral and molecular changes following sexual abuse are not yet fully understood.

Although childhood sexual abuse often co-occurs with other types of adversites,^30^ it still contributes independently to the risk of developing anxiety and depression,^4, 31^ two leading causes of global health burden according to the World Health Organization.^32, 33^ However, not all sexual abuse victims develop anxiety or depression,^34, 35^ indicating individual differences in susceptibilities to these symptoms, some of which may be genetically influenced.^36, 37^ The genetic contribution to symptom outcomes among people victimized by sexual abuse has been sparsely studied, with no replicable genetic association detected to date.^38, 39, 40^ The lack of robust associations poses one of the biggest challenges in contemporary psychiatric genetics as behavioral traits are complex and loci behind them explain only a small fraction of the phenotypic variance.^41^ Although it proves difficult to collect large samples of sexual abuse victims to achieve the needed power to detect small effect sizes, one approach to elucidating the genetics of psychiatric outcomes in this group could be to use animal models as a candidate gene-generating tool. Clearly, no animal model of sexual abuse exists. However, childhood sexual abuse can be broadly characterized as early-life stress, for which several animal models are available. Early-life stress can be modeled in animals in the form of compromised maternal care, such as postnatal long maternal separation (LMS; 3 h each day after birth for 2 weeks). The separation produces more persistent anxiety- and depression-like behaviors, as well as concomitant endocrine and neurochemical changes when LMS offspring are compared with brief maternal separation (BMS; 10–15 min each day after birth mimicking naturally occurring separation from the mother), rather than to non-handled offspring (NH; left undisturbed with their mother).^42, 43, 44^

In the present translational study, we used this well-known rodent early-life stress model to identify genes revealing differential expression in hippocampus and/or pineal gland across early-life stress conditions. Owing to parallels in the stress pathology, we hypothesized that the human orthologs of the identified genes would show associations with depression and anxiety symptomatology in human adults who had experienced sexual abuse early in life.

## Materials and methods

### Animal study

This animal study was part of a larger project examining the effects of both early- and later-life stress conditions on behavior and modulation of brain gene expression.^43, 44^ All the procedures were performed according to guidelines of the Norwegian Animal Research Authority (Permit Number: 07/9421-2007025) and conducted in accordance with The European Convention for the protection of Vertebrate Animals used for Experimental and other scientific purposes (18 March 1986). The same personnel handled the animals throughout the study.

#### Animal breeding and early- and later-life stress

For mating, two females were housed with one male rat of Wistar strain (NTac:WH, Taconic, Silkeborg, Denmark). Ten females delivered 120 offspring and the day of birth was designated postnatal day (PND) 0. The litter size ranged from 4 to 15. Cross-fostering was performed within 24 h after birth to equalize the litter size to 12 offspring per litter. The mother and litter were housed in individually ventilated cages (type IV, Tecniplast, Buggugitate, Italy) with an ambient temperature of 22±1 °C and air humidity of 52±2%. The light and dark cycles were 12:12 h with lights gradually increasing/decreasing at 0600 h and 1800 h and fully on/off at 0700 h and 1900 h. Breeding diet (RM3, Special Diets Services, Witham, Essex, UK) and water were available *ad libitum* and replenished once a week. Bedding (Bee Kay Bedding, Scanbur, Karlslunde, Denmark) was changed once a week, except during PND 0–14.

The pups were exposed to maternal separation or a non-handling condition daily from PND 2 to 14. The mother was first moved to a separate cage with food and water *ad libitum*. The litter was then moved to a different room with a cage containing chopped wood bedding and soft paper. A heating lamp provided a stable temperature (PND 2–7: 32–34 °C, PND 8–14: 28–30 °C). The mother and her offspring were reunited in the reverse order. LMS involved 180 min-long separation, whereas BMS lasted for 10 min, both starting at 0900 h. The offspring in the NH condition were left undisturbed with their mother (for more details, see ref. 45). Four to five offspring of the same sex and litter were housed in the same cage at weaning, PND 22. At PND 55–60, all the animals underwent a surgical procedure for implantation of transmitter (Physiotel, Data Sciences International, St. Paul, MN, USA) for continuous wireless recording of sleep and temperature rhythms, as previously described.^43, 44^ In brief, the animals were anesthetized with subcutaneous injection of a mixture of fentanyl 0.277 mg kg^−1^, fluanizone 8.8 mg kg^−1^ and midazolam 2.5 mg kg^−1^ (Hypnorm, Janssen, Beerse, Belgium; Dormicum, Roche, Basel, Switzerland; Midazolam Actavis, Actavis, Parsippany-Troy Hills, NJ, USA) and the transmitters were placed in subcutaneous pockets in the dorsomedial lumbar region. The animals were housed individually in individually ventilated type III cages thereafter and allowed to recover for 14 days before entering the experiment. All the animals were anesthetized with pentobarbital and decapitated. The brain was rapidly separated from the skull and the brain regions (ventral hippocampus and pineal gland) dissected on an ice-cold glass dish, aliquoted into Eppendorf tubes (Horsholm, Denmark) and stored at −80 °C until analysis.

Male rats (*n*=60) from each early-life condition were subdivided into subgroups of later-life stress; a chronic mild stress and a control group, randomly and balanced between the litters. The 4-week chronic mild stress paradigm consisted of unpredictable exposure to a variety of mild stressors, see further details in ref. 45. The control group were given standard animal care in a separate room.

#### RNA preparation, labeling and microarray hybridization

The hippocampal tissue was extracted from 18 NH, 18 BMS and 24 LMS offspring, whereas the pineal gland tissue was extracted from 12 NH, 9 BMS and 15 LMS offspring. The tissues were harvested randomly and balanced between the early-life condition, the later-life condition and between the litters. About 10 mg tissue from each sample was homogenized using the TissueLyser tissue disruptor (QIAGEN, Hilden, Germany) for 2 × 30 s at 20 000 r.p.m. Total RNA was extracted using the ABI PRISM 6100 Nucleic Acid Prep Station (Applied Biosystems, Foster City, CA, USA). The amount and quality of total RNA was measured using the NanoDrop Spectrophotometer (NanoDrop Technologies, Wilmington, DE, USA) and the Agilent 2100 Bioanalyzer (Santa Clara, CA, USA). All the samples had an RNA integrity number above 7.5.^46^ All microarray analyses were performed using the Illumina Whole Genome Expression Bead Chips (Illumina, San Diego, CA, USA). Total RNA (500 ng) from hippocampus and pineal gland was reverse transcribed, amplified and biotin-labeled using the Illumina Total Prep RNA amplification kit (Ambion, Huntingdon, UK). Biotin-labeled complementary RNA (750 ng) was hybridized to Illumina RatRef-12 Expression Bead Chips (Illumina) according to the manufacturer's instructions. These chips contain 22 523 probes, representing 22 228 rat genes, selected primarily from the NCBI RefSeq database (release 16). Following hybridization, the Bead Chips were washed and stained with Streptavidin-Cy3 (Thermo Fisher Scientific, Waltham, MA, USA). Fluorescent signal detection was performed by the iScan reader (Illumina) and the resulting images were processed by Genome Studio Software v2009.1 (Illumina). The signal intensities were imported into the J-Express 2012 software (Molmine, Bergen, Norway),^47^ where inter-array quantile normalization and base 2 logarithmic transformation were performed to minimize the technical artifacts (for example, RNA extraction, labeling and hybridization) and to obtain a normal distribution, respectively. The investigators were blinded to the group allocation during the analyses.

#### Analysis of gene expression

Global trends in the data were examined by correspondence analysis.^48^ In the correspondence analysis plot, the microarray data for genes and samples are projected onto a two-dimensional plane defined by the first and second principal components. The samples that are close together in the plot have a more similar global gene expression than those that are further apart.

Identification of microarray probes that differed significantly in expression level (that is, hybridization signal intensity) in the hippocampus and/or pineal gland between the different early-life conditions was carried out by significance analysis of microarrays (SAM),^49^ comparing signal intensities of all probes across early-life conditions within a specific brain region. Separate SAM analyses were thus performed within the hippocampus and pineal gland, respectively; LMS vs NH, LMS vs BMS and BMS vs NH. To minimize the number of false positives, the SAM analysis threshold was set to a *q*-value of 0 (see ref. 50).

To identify genes that may be affected as a result of adverse life events, we used a combination of statistical significance of differential expression, visual inspection of gene expression profile across individual samples and potential involvement of candidate genes in neurological development and/or disorders. More specifically, the list of differentially expressed genes resulting from the SAM analysis was subjected to visual inspection of individual gene expression profiles, aiming to eliminate false positive findings that may have arisen as a result of low signal levels and technical artefacts. This trimmed list was subsequently screened for genes with known or suggested involvement in neurological development and/or disorders, by manual inspection of gene annotation such as gene ontology and pathways, as well as literature searches.

### Human genetic association study

#### Participants

Sexually abused individuals were recruited from two different sources: (1) support centers for sexual abuse victims (2) a representative sample of the Norwegian population aged 18–80 years.^3, 51^ The recruitment of participants was conducted in line with ethical principles specified in the Declaration of Helsinki, including the ensuring of informed consent to participate in the study.

All respondents from the sexual abuse support centers were classified as sexually abused based on self-reports. From the general population of Norway aged 18–80 years, a random sample of 1450 men and women were invited to participate in a survey assessing unwanted sexual experiences, of which 703 (48.7%) responded. Subsequently, a saliva collection kit was sent to those who agreed to provide a saliva sample (*n*=306). Unwanted sexual experiences were classified according to the sexual abuse categorization provided by the Norwegian criminal code, which differentiates between unwanted sexual behaviors (for example, sexual exposure or other sexual behaviors not involving physical contact), unwanted sexual acts (for example, sexual touching and fondling) and unwanted intercourse (for example, penetration of fingers/penis/object into victims anus/vagina/mouth). To reduce the likelihood of falsely classifying people as sexually abused, only those reporting unwanted sexual intercourse or acts were included in the study (see Supplementary Table 1 for an overview of the items used to assess these experiences). In the representative population study, a check list assessed the respondents' exposure to multiple types of unwanted sexual acts and intercourse. Their age the first time these incidents took place was assessed separately for each item. In the sample of support center users, age at first abusive incident was assessed using an open-ended question.

In total, 710 recruited individuals (537 from the support centers and 173 from the general population) were classified as sexually abused (aged 17 to 73 years; Mean =41.6, s.d.=13.1, 93.1% female). Four hundred and three participants provided DNA samples (306 from the support centers and 97 from the general population; see Supplementary Figure 3 for a flowchart displaying this selection process). Mean age at the first abusive incident was 6.5 years (s.d.=3.9 years) in the sample of support center users, and 14.8 years (s.d.=4.1 years) for sexual abuse involving intercourse in the representative population sample.

Each participant provided a DNA sample collected with the OG-100 saliva kit (Oragene, DNA Genotek, Ottawa, ON, Canada) and a standard Oragene protocol was applied to perform DNA extraction. The study was approved by the Regional Committee for Medical and Health Research Ethics of Western Norway (approval number 264.08).

#### Measures of anxiety and depression symptoms

Symptoms of anxiety and depression were assessed using the Hospital Anxiety and Depression Scale (HADS).^52^ Cronbach's α for the total scale in the current sample was 0.91. To categorize symptoms of anxiety and depression from the HADS, a person-driven approach (fuzzy clustering) was applied.^53^ Fuzzy clustering was performed in the R software, using FANNY algorithm.^54^ Each individual was assigned to one of the following clusters: ‘No symptoms' (scoring low on both anxiety and depression scales), ‘Anxiety only' (scoring high on anxiety scale and low on depression scale), ‘Depression only' (scoring high on depression scale and low on anxiety scale) and ‘Comorbid symptoms' (scoring high on both anxiety and depression scales).

Apart from sexual abuse and anxiety and depression symptoms measures, the participants were also assessed for their current perception of social support using the Multidimensional Scale of Perceived Social Support questionnaire,^55^ which addresses perceived social support from friends, family and significant others. Cronbach's α for the scale was 0.93.

#### SNP selection and genotyping

SNPs (single-nucleotide changes in the DNA sequence with a minor allele frequency of at least 1%) of the candidate genes identified in the animal study were tagged in Haploview software;^56^ using HapMap CEU genotype data (release 28). The tagging was based on the SNPs passing the following criteria: minor allele frequency above 5%, no Mendelian errors, Hardy–Weinberg *P*-value below 0.01 and genotyping rate above 95%. Pairwise tagging algorithm was performed with *r*^2^ threshold above 0.8.

Genotyping of tagging SNPs was accomplished by MassArray iPlex (Sequenom, San Diego, CA, USA) system at CIGENE center for genotyping (University of Life Sciences, Ås, Norway). Genotyping quality control was implemented in PLINK and consisted of Hardy–Weinberg test (*P*<0.009) and genotyping rate above 95%.^57^ In addition, the variants' minor allele frequencies observed in our sample were compared with those reported for CEU population in the 1000 Genomes Project (pilot one).

### Statistical analyses

Before the assessment of genetic association, exploratory regression analyses were performed to determine possible confounders measured in this study. Thus, the effect of gender, age, recruitment source and perceived social support on the measured anxiety and depression symptoms was calculated. Genetic association between tagging SNPs and the anxiety and depression symptoms measure was then tested, adjusting for confounders revealing significant effects in the exploratory step. All modeling was done in the form of multinominal logistic regression. Correction for multiple testing was achieved by false discovery rate.^58^ The false discovery rate *q*-value below 0.05 was considered significant. All analyses were conducted in the R software.

The functional potential of polymorphisms revealing significant associations before false discovery rate correction was examined *in silico* in relation to the brain tissue as well as hippocampus specifically using HaploReg software, version 4.^59^ Given that we examined tagging SNPs, we extended our HaploReg evaluation to all SNPs in linkage disequilibrium with the examined ones (*r*^2^ >0.8).

Exploratory analysis revealed statistically significant effects of age, perceived social support and recruitment source (representative population vs support centers) on the anxiety and depression outcome measure. Subsequently, age, perceived social support and sample source were included as covariates in the final regression model testing for genetic association. We used the ‘No symptoms' group as the reference group.

## Results

### Animal study

#### Gene expression in the hippocampus and pineal gland of LMS, BMS and NH offspring

Global gene expression in the hippocampus and pineal gland of rats subjected to the three different early-life conditions was first studied by correspondence analysis. On the global level, within each brain region, no systematic differences in the brain gene expression could be observed between any of the different early-life conditions (Supplementary Figures 1 and 2).

**Figure 1:**
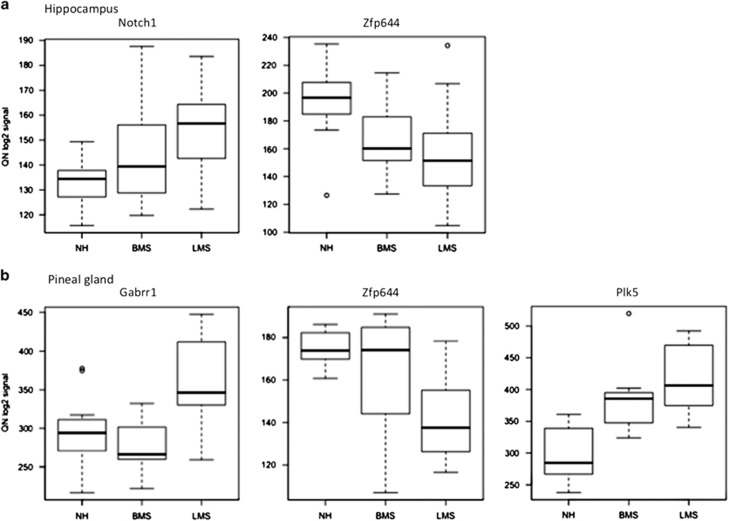
Box plots illustrating relative expression levels (quantile normalized, log2-transformed signal intensities) of *Notch1*, *Zfp644*, *Gabrr1* and *Plk5* in hippocampus (**a**) and/or pineal gland (**b**) of rat offspring experiencing long (LMS), brief (BMS) or no (NH) maternal separation. The box plots indicate the median of the distribution (thick black line), 75th percentile (upper edge of box), 25th percentile (lower edge of box), 95th percentile (upper edge of vertical line), 5th percentile (lower edge of vertical line) and the outlier points (above and below vertical lines).

Gene level differential gene expression after early-life stress was examined by SAM. Very few genes displayed significant differential expression between the different early-life stress conditions. Within the hippocampus, seven, three and two genes were significantly differentially expressed between LMS and NH, LMS and BMS, as well as BMS and NH offspring, respectively (Table 1). Within the pineal gland, seven genes were significantly differentially expressed between LMS and NH offspring, and two genes between LMS and BMS offspring (Table 1). A few genes showed significant differences across several conditions, such as *Plk5* being upregulated in the pineal gland of both LMS and BMS offspring compared with NH offspring, and *Zfp644* being downregulated in LMS offspring compared with NH offspring in both hippocampus and pineal gland (see Figure 1 and Table 1). Several genes showed similar tendencies, although not significantly, in additional comparisons (see Supplementary Table 2). Furthermore, as illustrated in Figure 1, among the BMS brain samples, many of the differentially expressed genes displayed intermediary expression levels, falling somewhat in the middle between those observed in the brain samples of NH and LMS offspring (also see Supplementary Table 2).

By visual inspection of individual gene expression profiles and gene annotation, we selected four genes showing robust differential expression among animals subjected to different early-life conditions, as well as having potential relevance for neurological development or disorders for further analysis in the human association study: *Notch1*, *Gabrr1*, *Plk5* and *Zfp644* (Table 1).

### Human genetic association study

#### Anxiety and depression symptoms

Fuzzy clustering of HADS measures identified three main clusters (Table 2). Cluster 1 was characterized by low scores on both the anxiety and depression subscales, and was thus labeled as ‘No symptoms' group. Cluster 2 revealed high scoring on the anxiety subscale and low scoring on the depression subscale, and was labeled as ‘Anxiety only' group. Cluster 3 was labeled as ‘Comorbid symptoms' group, based on scoring high on both the anxiety and depression subscales. A ‘depression only' cluster was not observed.

#### SNP selection and genotyping

We identified the four human orthologs of the selected candidate genes from the rat study: *NOTCH1*, *PLK5*, *ZNF644* and *GABRR1*. Overall, 47 SNPs were determined as tag SNPs for them. One SNP failed the multiplex design and two SNPs failed the Hardy–Weinberg test, leaving 44 SNPs available for the analyses. In total, 361 participants (269 from the support centers for sexual abuse survivors and 92 from the general population) were successfully genotyped (Supplementary Figure 3).

#### Associations of SNPs with anxiety and depression symptoms

Significant associations were noted for four SNPs in the *GABRR1* gene and one SNP in the *NOTCH1* gene when comparing the ‘No symptoms' to the ‘Anxiety only' group. However, none of these remained significant after correction for multiple testing (Table 3a). When comparing ‘No symptoms' group to the ‘Comorbid symptoms' group, significant effects were found for four SNPs in the *NOTCH1* gene (Table 3b). Two of these SNPs, rs3013302 and rs11145770, survived the correction for multiple testing. No significant effects were found in *ZNF644* and *PLK5* genes. The association results of all the examined SNPs are summarized in Supplementary Table 3.

HaploReg analyses showed regulatory potential for SNPs within the *NOTCH1* gene, including those in high linkage disequilibrium with rs3013302 and rs11145770, with promoter and enhancer activity in fetal brain and several brain regions, including the hippocampus. In addition, these SNPs also revealed eQTL (expression quantitative trait locus) activity in relation to the following genes: *NALT1* (RP11-611D20.2 transcript), *INPP5E*, *CARD9*, *PMPCA, SNAPC4* and *LOC286254*. In the *GABRR1* gene, only one SNP—rs453503—displayed promoter activity in the brain, but not in the hippocampus. Similarly to *NOTCH1*, variants in *GABRR1* also possessed eQTL function as reported in arterial tissue.^60^ All HaploReg results are summarized in Supplementary Table 4.

## Discussion

In this translational study, we aimed to examine the genetic contribution to anxiety and depression symptoms among sexual abuse victims using an animal experimental study as a candidate gene-generating tool. Among a list of 20 differentially expressed genes in the brains of rats exposed to different early-life conditions, we selected four candidate genes based on their level and robustness of differential gene expression, as well as potential relevance for neural development or disorders. These candidate genes were then tested for their association with anxiety and depression symptoms in a sample of human adults who had experienced sexual abuse early in life.

The main finding of the present study was the implication of *NOTCH1* as a candidate gene for the pathomechanisms following early-life stress that may be relevant for the development of anxiety and depression in humans who experienced sexual abuse. We observed increased expression of this gene in the hippocampus of rats exposed to long maternal separations early in life compared with those who were left undisturbed with their mother (Figure 1, Table 1). *NOTCH1* also showed associations with anxiety and depression symptoms in our sample of sexual abuse victims. Specifically, minor alleles of two *NOTCH1* tag SNPs—rs3013302 and rs11145770—were associated with higher likelihood of displaying comorbid anxiety and depression symptoms compared with not displaying these symptoms in a sample of sexual abuse victims (Table 3b).

The *NOTCH1* gene encodes the NOTCH1 receptor, a member of the Notch transmembrane protein family.^61^ Its expression is documented in several areas of the adult brain, including the hippocampus in both humans and mice.^62, 63^ Functionally, the Notch signaling pathway influences cell fate decisions in many developmental processes,^61^ and the *Notch1* gene has been reported to have important regulatory roles in hippocampal neurogenesis in animal studies.^64, 65^ Importantly, expression of *NOTCH1* has been found to be influenced by glucocorticoids,^66, 67^ hormones elevated in response to stress. Moreover, cross-talk between glucocorticoids and the Notch signaling pathway has been demonstrated in human hippocampal cells^68^ as well as in other cell types.^66, 67, 69^ It has also become increasingly evident that newly produced hippocampal neurons may have a direct role in regulating the stress response.^70, 71^ In addition, the Notch signaling pathway has been implicated in the development of depression symptoms in a previous study.^72^ Moreover, as our comparison group consisted of sexual abuse victims who did not report symptoms of anxiety and depression, *NOTCH1* may also be postulated to have a role in the neurobiology of resilience to these symptoms among sexual abuse victims.

Stress-induced changes in hippocampal neurogenesis have long been believed to have a key role in the etiology of depression and anxiety.^73, 74^ A number of studies report that stress is both a suppressor of hippocampal neurogenesis and a significant precipitating factor in the development of depression and anxiety.^73, 74, 75, 76^ In addition, studies suggest that hippocampal neurogenesis may mediate the effect of antidepressants.^77, 78^ These findings correspond with neuroimaging studies showing hippocampal atrophy observed in people who have experienced stressful events, including sexual abuse,^10, 11, 12, 13, 79^ as well as in people with depression and anxiety disorders.^80, 81, 82^ In addition, hippocampal neurogenesis has been found to mediate the association between early-life stress and depression longitudinally.^79^ Thus, our observation of Notch1 expression change following exposure to early-life stress in rats as well as the association of *NOTCH1* tag SNPs with anxiety and depression symptoms in victims of sexual abuse is in line with the neurogenesis hypothesis of affective and anxiety disorders^73, 74, 75, 76, 83, 84^

Both tag SNPs of *NOTCH1* gene revealing associations with anxiety and depression symptoms in this study, as well as variants in high linkage disequilibrium with them, showed regulatory potential by displaying both enhancer and promoter properties in brain tissue, including hippocampus (Supplementary Table 4). Moreover, these SNPs also exhibit eQTL activity related to long noncoding RNA genes (*LOC286254, NALT1*) and genes involved in signal transduction (*INPP5E, CARD9*), mitochondrial processing (*PMPCA*) and nuclear RNA activation (*SNPC4*). It has recently been observed that SNPs previously associated with neurological and psychiatric conditions may be highly concentrated in the regions of long noncoding RNA genes.^85^ Furthermore, the apparent lack of exonic polymorphisms among genome-wide significant associations of psychiatric disorders may suggest that alterations in gene expression rather than protein structure could be the molecular mechanism leading to these conditions.^86, 87^ Such eQTL effect of SNPs in *NOTCH1* gene may also indicate that a pathway other than NOTCH1 could underlie the current findings.

Apart from *Notch1*, we also noted significant changes in gene expression of *Gabbr1*, *Plk5* and *Zfp644* in the rodent model (Table 1). Similar to *NOTCH1*, the orthologs of these genes have previously been implicated in both neurogenesis and stress.^88, 89, 90, 91^ However, only *GABRR1* revealed nominal association with anxiety and depression symptoms in our sample of sexually abused individuals (Table 3a).

*GABRR1* gene encodes the ionotropic GABA_A_ receptor of gamma aminobutyric acid (GABA), the main inhibitory neurotransmitter in the central nervous system.^92^ The activation of GABA_A_ has been reported to inhibit neuronal activity,^93^ making this receptor the target for a vast number of psychoactive drugs, including anxiolytics (anti-anxiety) and antidepressants.^94, 95^ Indeed, considerable evidence from a number of studies suggests that GABA_A_ has an important role in the pathogenesis of both anxiety and depression.^96^ It has also been shown that conflict stress can alter the expression of GABA receptor subunits in rodents, which, in turn, may lead to dramatic changes in its function.^97^ Moreover, GABAergic mechanisms have been proposed as possible mediators of the interplay between aversive memories and stress endocrinology.^96^

The expression of *Plk5* and *Zfp644* was altered in our rodent model of early-life stress (Table 1). *Plk5* encodes polo-like kinase 5, while *Zfp644* encodes zinc finger protein 644. *Plk5* is mostly expressed in the brain, where it is involved in the regulation of neuritic processes.^88^ Paralogs of this gene have been implicated in stress response pathways.^98^ *Zfp644* is a ubiquitously expressed transcription factor, whose function has not yet been substantially characterized. So far, it has been implicated in eye development.^99^ Although the tag SNPs in orthologs of these genes did not reveal associations with anxiety and depression symptoms in the current sample of sexually abused individuals, their altered expression following early-life stress in rats indicates their relevance as interesting candidate genes for further exploration of health outcomes following stressful events, especially as molecular characterization of these genes is sparse.

Some limitations of the present studies should be noted. The small number of litters may explain the lack of more statistical power in terms of detecting more candidate genes. Also, the animal study did not examine active maternal behavior (licking and grooming activity) during the postnatal period. Active maternal care is important for normal neuronal development. Adding information of maternal behavior could have provided important indications as to whether differences in gene expression observed in adult LMS and NH offspring may be associated with low/high levels of maternal licking and grooming. Given our modest sample size of sexually abused individuals, one major constraint of an association study is its modest power that may have prevented us from detecting signals of small effects sizes. The mood phenotypes in our sample of sexually abused individuals were derived from self-reports, whose clinical validity may be considered uncertain. However, HADS scores have been shown to be a good reflection of clinical anxiety and depression.^100^ Given the lack of information on ethnicity in the present study, we cannot rule out potential biases introduced by population stratification in our genetic association findings. However, such bias is unlikely as the minor allele frequencies of examined SNPs in our sample did not deviate significantly from the Northern European sample from Utah (CEU) of the 1000 Genomes Project, suggesting that our Norwegian sample reflects a European population overall. It should also be noted that we examined tag SNPs only, allowing us to capture significant loci rather than specific polymorphisms. Finally, our comparison group was shared between analyses of anxiety only and comorbid conditions. This may have biased our false discovery rate correction. Thus, our findings implicating *NOTCH1* gene in susceptibility to anxiety and depression among sexual abuse victims should be further examined in larger samples, using clinically validated mood phenotypes.

In conclusion, our results revealed a number of candidate genes with differential expression in brain regions following early-life stress in rats (Table 1). Furthermore, our results implicate the *NOTCH1* system in the susceptibility to comorbid anxiety and depression symptoms in a sample of sexually abused individuals. Although further investigations clearly are needed to validate and elucidate the exact role of the Notch signaling pathway in the pathology of stressful events, the associations observed in our study of sexually abused individuals together with the differential expression pattern in rats support the notion that *NOTCH1* could be involved in the liability and resilience to such pathology.

## Figures and Tables

**Table 1:** Genes with significant (*q*=0) differential expression in pineal gland and/or hippocampus between animals subjected to LMS, BMS and NH controls

*Probe ID*	*Gene ID*	*Gene symbol*	*Gene name*	*Fold difference*^a^
*Pineal gland*				
* BMS vs NH*				
* *ILMN_1375780	303439	*Maf*	Monocyte to macrophage differentiation-associated	1.23
* *ILMN_1352420	314627	***Plk5***	Polo-like kinase 5	1.30
* LMS vs NH*				
* *ILMN_1369757	305127	***Zfp644***	Zinc finger protein 644	−1.24
* *ILMN_1354493	25338	*Ninj1*	Ninjurin 1	−1.19
* *ILMN_1368424	303702	*Engase*	Endo-beta-*N*-acetylglucosaminidase	−1.15
* *ILMN_1373511	116745	*Kcnh6*	Potassium voltage-gated channel, subfamily H (eag-related), member 6	1.27
* *ILMN_1352420	314627	***Plk5***	Polo-like kinase 5	1.40
* *ILMN_1376353	311872	*Zbtb43*	Zinc finger and BTB domain containing 43	1.43
* *ILMN_1372167	313644	*Rap1ga1*	RAP1, GTPase activating protein 1	2.00
* LMS vs BMS*				
* *ILMN_1356838	308060	*Ccdc127*	Coiled-coil domain containing 127	1.25
* *ILMN_1373838	29694	***Gabrr1***	Gamma-aminobutyric acid (GABA) receptor, rho 1	1.32
* *ILMN_1349159	498433	*Psme4*	Proteasome activator subunit 4	−1.44

*Hippocampus*				
* LMS vs NH*				
* *ILMN_1357880	64347	*Sncg*	Synuclein, gamma (breast cancer-specific protein 1)	−1.60
* *ILMN_1373010	362484	*Plekhf2*	Pleckstrin homology domain containing, family F (with FYVE domain) member 2	−1.39
* *ILMN_1373132	362134	*Mmadhc*	Methylmalonic aciduria and homocystinuria, cblD type	−1.41
* *ILMN_1373950	362685	*Gpatch11*	G patch domain containing 11	−1.30
* *ILMN_1369757	305127	***Zfp644***	Zinc finger protein 644	−1.27
* *ILMN_1370841	309391	*Cox15*	COX15 homolog, cytochrome c oxidase assembly protein	1.09
* *ILMN_1359640	25496	***Notch1***	Notch gene homolog 1	1.16
* LMS vs BMS*				
* *ILMN_1356902	29131	*Cartpt*	CART prepropeptide	−1.24
* *ILMN_1650165	295264	*Mllt11*	Myeloid/lymphoid or mixed-lineage leukemia; translocated to 11	−1.32

Abbreviations: BMS, brief maternal separation; LMS, long maternal separation; NH, non-handled.

a Positive or negative fold difference indicates up- or downregulation of probe in first vs second group, for example, in BMS vs NH.

Group comparison is indicated in italics. Genes selected for human genetic association study are highlighted in bold.

**Table 2:** Means and standard deviations of HADS subscale scores in the three observed clusters

*Symptoms*	*No symptoms group;* *Mean (s.d.)*	*Anxiety-only group;* *Mean (s.d.)*	*Comorbid symptoms group;* *Mean (s.d.)*
HADS-anxiety	3.29 (1.63)	8.43 (2.14)	13.13 (2.97)
HADS-depression	1.56 (1.72)	3.48 (2.09)	8.82 (3.27)

Abbreviation: HADS, Hospital Anxiety and Depression Scale.

Interpretation of HADS scoress: ⩾8: possible, and ⩾11: probable clinically significant anxiety/depression.^52^

**Table 3a:** Association results of the nominally significant SNPs in the human study

*Gene*	*SNP*	*Chr.*	*Position (GRCh38)*	*‘No symptoms' vs ‘Anxiety only'*		
				*OR (95% CI)*	P*-value*	q*-value*
*GABRR1*	rs9342185	6	89204419	0.44 (0.25–0.78)	0.005	0.215
*GABRR1*	rs4707529	6	89208843	0.47 (0.26–0.86)	0.015	0.241
*GABRR1*	rs7758893	6	89206922	0.53 (0.30–0.92)	0.025	0.241
*GABRR1*	rs453503	6	89190880	0.52 (0.29–0.93)	0.028	0.241
*NOTCH1*	rs11145770	9	136532614	1.79 (1.10–2.89)	0.018	0.241

Abbreviations: 95% CI, 95% confidence interval; OR, odds ratio; SNP, single-nucleotide polymorphism.

False discovery rate *q*-value <0.05.

The full table of results is presented in Supplementary Table 3.

**Table 3b:** Association results of the nominally significant SNPs in the human study

*Gene*	*SNP*	*Chr.*	*Position (GRCh38)*	*‘No symptoms' vs ‘Comorbid symptoms'*		
				*OR (95% CI)*	P*-value*	q*-value*
*NOTCH1*	rs11145770	9	136532614	2.21 (1.35–3.61)	0.002	0.043^a^
*NOTCH1*	rs3013302	9	136537422	2.15 (1.32–3.49)	0.002	0.043^a^
*NOTCH1*	rs13301342	9	136499893	0.36 (0.16–0.77)	0.009	0.097
*NOTCH1*	rs13290979	9	136531182	1.92 (1.18–3.15)	0.009	0.097

Abbreviations: 95% CI, 95% confidence interval; OR, odds ratio; SNP, single-nucleotide polymorphism.

a False discovery rate *q*-value <0.05.

The full table of results is presented in Supplementary Table 3.
