# Supplementary material for: Implication of NOTCH1 gene in susceptibility to anxiety and depression among sexual abuse victims
Source: Transl Psychiatry. 2016 Dec 13;6(12):e977–. doi: 10.1038/tp.2016.248 (PMC5290341; doi:10.1038/tp.2016.248)
Supplement: Supplementary Table 1 [file tp2016248x1.docx]

| ***Unwanted sexual acts*** | Touching/fondling of genitals/breasts |
| --- | --- |
|  | Repeated movements mimicking intercourse towards one’s body |
| ***Unwanted sexual intercourse*** | Been forced to masturbate others |
|  | Penetration with fingers in vagina or anus |
|  | Penetration with object in vagina or anus |
|  | Penetration with genitals in mouth, vagina or anus |
|  | Been forced to sexual intercourse by means of violence or threatening behavior |
|  | Been forced to sexual intercourse while unconscious or incapable of resisting it |
|  | Been pressured to sexual intercourse in the absence of violence or threats |
| *Note*: Response alternatives for each item were “Yes” and “No”. When confirming an item, respondents were asked to indicate their age the first time the offence happened. | |

Supplementary Table 1. Items used to assess sexual abuse (unwanted sexual acts and unwanted sexual intercourse) in the representative population sample

*“Have you experienced any of the following sexual offenses?”*
