# Supplementary material for: Implication of NOTCH1 gene in susceptibility to anxiety and depression among sexual abuse victims
Source: Transl Psychiatry. 2016 Dec 13;6(12):e977–. doi: 10.1038/tp.2016.248 (PMC5290341; doi:10.1038/tp.2016.248)
Supplement: Supplementary Table 3 [file tp2016248x3.docx]

Supplementary Table 3. Summary of association results of all tagging SNPs examined in the sample of sexual abuse victims.

A) Comparison of “No symptoms” versus “Anxiety only” groups.

| **Gene** | **SNP** | **Chr.** | **Position (Build GRCh38)** | **“No symptoms” vs “Anxiety only”** | | |
| --- | --- | --- | --- | --- | --- | --- |
|  |  |  |  | **OR (95% CI)** | **p-value** | **q-value** |
| GABRR1 | rs9342185 | 6 | 89204419 | 0.44 (0.25 - 0.78) | 0.005 | 0.215 |
| GABRR1 | rs4707529 | 6 | 89208843 | 0.47 (0.26 - 0.86) | 0.015 | 0.241 |
| GABRR1 | rs7758893 | 6 | 89206922 | 0.53 (0.30 - 0.92) | 0.025 | 0.241 |
| GABRR1 | rs453503 | 6 | 89190880 | 0.52 (0.29 - 0.93) | 0.028 | 0.241 |
| NOTCH1 | rs11145770 | 9 | 136532614 | 1.79 (1.10 - 2.89) | 0.018 | 0.241 |
| NOTCH1 | rs3013302 | 9 | 136537422 | 1.57 (0.98 - 2.51) | 0.06 | 0.373 |
| NOTCH1 | rs13290979 | 9 | 136531182 | 1.58 (0.98 - 2.54) | 0.062 | 0.373 |
| GABRR1 | rs13215160 | 6 | 89208667 | 1.86 (0.94 - 3.69) | 0.077 | 0.373 |
| GABRR1 | rs422751 | 6 | 89198154 | 1.80 (0.94 - 3.44) | 0.078 | 0.373 |
| GABRR1 | rs13215029 | 6 | 89208623 | 1.69 (0.91 - 3.11) | 0.094 | 0.404 |
| NOTCH1 | rs13301342 | 9 | 136499893 | 0.59 (0.29 - 1.20) | 0.146 | 0.526 |
| NOTCH1 | rs3124999 | 9 | 136501021 | 1.42 (0.88 - 2.26) | 0.147 | 0.526 |
| GABRR1 | rs368873 | 6 | 89183443 | 0.70 (0.43 - 1.15) | 0.159 | 0.526 |
| GABRR1 | rs6902106 | 6 | 89216021 | 1.39 (0.85 - 2.27) | 0.19 | 0.559 |
| GABRR1 | rs12200969 | 6 | 89217247 | 1.38 (0.85 - 2.24) | 0.195 | 0.559 |
| NOTCH1 | rs2229971 | 9 | 136513480 | 1.32 (0.83 - 2.11) | 0.243 | 0.609 |
| GABRR1 | rs439912 | 6 | 89197296 | 1.32 (0.81 - 2.14) | 0.269 | 0.609 |
| GABRR1 | rs3734201 | 6 | 89178724 | 1.29 (0.82 - 2.03) | 0.269 | 0.609 |
| GABRR1 | rs1796743 | 6 | 89179025 | 0.77 (0.48 - 1.23) | 0.269 | 0.609 |
| GABRR1 | rs2183797 | 6 | 89204468 | 0.78 (0.48 - 1.26) | 0.301 | 0.631 |
| GABRR1 | rs9353653 | 6 | 89211904 | 0.79 (0.50 - 1.25) | 0.308 | 0.631 |
| GABRR1 | rs2297389 | 6 | 89205787 | 1.42 (0.69 - 2.93) | 0.343 | 0.670 |
| PLK5 | rs2256653 | 19 | 1526540 | 1.24 (0.76 - 2.01) | 0.389 | 0.722 |
| GABRR1 | rs11754862 | 6 | 89201968 | 1.30 (0.70 - 2.40) | 0.403 | 0.722 |
| NOTCH1 | rs3812609 | 9 | 136514440 | 0.77 (0.39 - 1.51) | 0.443 | 0.762 |
| PLK5 | rs2292455 | 19 | 1526614 | 1.17 (0.75 - 1.85) | 0.489 | 0.809 |
| PLK5 | rs10853953 | 19 | 1533738 | 1.20 (0.68 - 2.10) | 0.527 | 0.818 |
| GABRR1 | rs881293 | 6 | 89208084 | 0.87 (0.54 - 1.40) | 0.565 | 0.818 |
| GABRR1 | rs407221 | 6 | 89180656 | 1.15 (0.70 - 1.89) | 0.574 | 0.818 |
| NOTCH1 | rs3812605 | 9 | 136508650 | 1.12 (0.72 - 1.73) | 0.613 | 0.818 |
| NOTCH1 | rs3124599 | 9 | 136509318 | 0.79 (0.30 - 2.04) | 0.623 | 0.818 |
| NOTCH1 | rs2229974 | 9 | 136497184 | 0.90 (0.58 - 1.40) | 0.639 | 0.818 |
| GABRR1 | rs2150817 | 6 | 89203801 | 1.12 (0.69 - 1.81) | 0.646 | 0.818 |
| PLK5 | rs2656864 | 19 | 1530034 | 1.11 (0.71 - 1.76) | 0.647 | 0.818 |
| PLK5 | rs2656865 | 19 | 1530061 | 1.10 (0.66 - 1.82) | 0.715 | 0.878 |
| GABRR1 | rs12198870 | 6 | 89200996 | 1.15 (0.45 - 2.94) | 0.774 | 0.911 |
| GABRR1 | rs407206 | 6 | 89185249 | 1.07 (0.67 - 1.70) | 0.784 | 0.911 |
| PLK5 | rs11084901 | 19 | 1535946 | 0.97 (0.61 - 1.54) | 0.897 | 0.993 |
| GABRR1 | rs2297391 | 6 | 89205685 | 1.04 (0.51 - 2.11) | 0.911 | 0.993 |
| GABRR1 | rs9444675 | 6 | 89207124 | 0.98 (0.62 - 1.56) | 0.927 | 0.993 |
| NOTCH1 | rs6563 | 9 | 136494732 | 0.99 (0.63 - 1.54) | 0.956 | 0.993 |
| PLK5 | rs2292449 | 19 | 1525308 | 0.66 (0.40 - 1.08) | 0.99 | 0.993 |
| NOTCH1 | rs3124596 | 9 | 136507052 | 1.00 (0.65 - 1.56) | 0.993 | 0.993 |
| *FDR q-value < 0.05 | | | | | | |

B) Comparison of “No symptoms” versus “Comorbid symptoms” groups.

| **Gene** | **SNP** | **Chr.** | **Position**  **(GRCh38)** | **“No symptoms” vs “Comorbid symptoms”** | | | |
| --- | --- | --- | --- | --- | --- | --- | --- |
|  |  |  |  | **OR (95% CI)** | **p-value** | | **q-value** |
| NOTCH1 | rs11145770 | 9 | 136532614 | 2.21 (1.35 - 3.61) | | 0.002 | 0.043 * |
| NOTCH1 | rs3013302 | 9 | 136537422 | 2.15 (1.32 - 3.49) | | 0.002 | 0.043 * |
| NOTCH1 | rs13301342 | 9 | 136499893 | 0.36 (0.16 - 0.77) | | 0.009 | 0.097 |
| NOTCH1 | rs13290979 | 9 | 136531182 | 1.92 (1.18 - 3.15) | | 0.009 | 0.097 |
| GABRR1 | rs6902106 | 6 | 89216021 | 1.61 (0.97 - 2.67) | | 0.067 | 0.532 |
| GABRR1 | rs13215029 | 6 | 89208623 | 1.77 (0.94 - 3.34) | | 0.075 | 0.532 |
| GABRR1 | rs9444675 | 6 | 89207124 | 0.67 (0.41 - 1.10) | | 0.112 | 0.532 |
| GABRR1 | rs3734201 | 6 | 89178724 | 1.43 (0.90 - 2.28) | | 0.13 | 0.532 |
| GABRR1 | rs12200969 | 6 | 89217247 | 1.48 (0.89 - 2.44) | | 0.13 | 0.532 |
| GABRR1 | rs13215160 | 6 | 89208667 | 1.74 (0.85 - 3.57) | | 0.133 | 0.532 |
| NOTCH1 | rs3124999 | 9 | 136501021 | 1.45 (0.89 - 2.35) | | 0.136 | 0.532 |
| GABRR1 | rs453503 | 6 | 89190880 | 0.66 (0.37 - 1.18) | | 0.159 | 0.562 |
| PLK5 | rs2292449 | 19 | 1525308 | 0.71 (0.43 - 1.17) | | 0.176 | 0.562 |
| NOTCH1 | rs3812609 | 9 | 136514440 | 0.62 (0.31 - 1.25) | | 0.183 | 0.562 |
| NOTCH1 | rs2229971 | 9 | 136513480 | 1.35 (0.83 - 2.19) | | 0.222 | 0.610 |
| GABRR1 | rs2297389 | 6 | 89205787 | 1.53 (0.74 - 3.17) | | 0.249 | 0.610 |
| GABRR1 | rs2150817 | 6 | 89203801 | 0.75 (0.45 - 1.23) | | 0.252 | 0.610 |
| GABRR1 | rs4707529 | 6 | 89208843 | 0.72 (0.40 - 1.29) | | 0.271 | 0.610 |
| GABRR1 | rs9342185 | 6 | 89204419 | 0.75 (0.44 - 1.28) | | 0.288 | 0.610 |
| GABRR1 | rs407206 | 6 | 89185249 | 0.77 (0.48 - 1.25) | | 0.294 | 0.610 |
| GABRR1 | rs11754862 | 6 | 89201968 | 1.39 (0.75 - 2.60) | | 0.298 | 0.610 |
| GABRR1 | rs422751 | 6 | 89198154 | 1.40 (0.72 - 2.74) | | 0.324 | 0.633 |
| GABRR1 | rs368873 | 6 | 89183443 | 0.80 (0.49 - 1.32) | | 0.385 | 0.685 |
| NOTCH1 | rs3124599 | 9 | 136509318 | 0.65 (0.24 - 1.79) | | 0.405 | 0.685 |
| GABRR1 | rs7758893 | 6 | 89206922 | 0.81 (0.47 - 1.39) | | 0.439 | 0.685 |
| PLK5 | rs11084901 | 19 | 1535946 | 0.83 (0.52 - 1.34) | | 0.448 | 0.685 |
| PLK5 | rs2256653 | 19 | 1526540 | 1.21 (0.74 - 1.99) | | 0.452 | 0.685 |
| NOTCH1 | rs3812605 | 9 | 136508650 | 1.18 (0.76 - 1.85) | | 0.462 | 0.685 |
| PLK5 | rs2292455 | 19 | 1526614 | 0.84 (0.52 - 1.34) | | 0.462 | 0.685 |
| PLK5 | rs2656864 | 19 | 1530034 | 1.17 (0.73 - 1.86) | | 0.518 | 0.742 |
| GABRR1 | rs1796743 | 6 | 89179025 | 0.87 (0.54 - 1.42) | | 0.578 | 0.753 |
| NOTCH1 | rs3124596 | 9 | 136507052 | 0.88 (0.56 - 1.39) | | 0.592 | 0.753 |
| GABRR1 | rs881293 | 6 | 89208084 | 1.14 (0.70 - 1.86) | | 0.599 | 0.753 |
| PLK5 | rs10853953 | 19 | 1533738 | 1.17 (0.66 - 2.07) | | 0.602 | 0.753 |
| PLK5 | rs2656865 | 19 | 1530061 | 1.14 (0.68 - 1.91) | | 0.617 | 0.753 |
| GABRR1 | rs2183797 | 6 | 89204468 | 1.13 (0.69 - 1.84) | | 0.63 | 0.753 |
| GABRR1 | rs12198870 | 6 | 89200996 | 1.13 (0.44 - 2.90) | | 0.793 | 0.923 |
| GABRR1 | rs2297391 | 6 | 89205685 | 1.08 (0.53 - 2.19) | | 0.843 | 0.940 |
| GABRR1 | rs407221 | 6 | 89180656 | 0.95 (0.57 - 1.60) | | 0.853 | 0.940 |
| NOTCH1 | rs2229974 | 9 | 136497184 | 0.97 (0.62 - 1.53) | | 0.9 | 0.966 |
| NOTCH1 | rs6563 | 9 | 136494732 | 0.98 (0.62 - 1.55) | | 0.93 | 0.975 |
| GABRR1 | rs439912 | 6 | 89197296 | 0.99 (0.60 - 1.63) | | 0.975 | 0.975 |
| GABRR1 | rs9353653 | 6 | 89211904 | 1.01 (0.63 - 1.62) | | 0.975 | 0.975 |
| *FDR q-value < 0.05 | | | | | | | |

**Supplementary Table 4. Summary of HaploReg results of the SNPs revealing association signals (p<0.05) prior to FDR correction.**

“Gene” refers to the Gene symbol, “SNP” refers to the dbSNP indetifier of the variant, “CHR” refers to chromosome on which the SNP is located, “BP” refers to basepair position where the SNP is located in hg38 Build, “OR” refers to odds ratio, “95% CI” refers to the confidence interval, “p-value” represents the association p-value in the respective comparisons and “FDR q-value” reveals the corresponding q-value of false discovey rate, “function” refers to the SNP location within a gene, “SNP in LD” presents a dbSNP ID of a varint in high linkage disequilibrium (LD, r^2^>0.8) with the examined variant and “LD(r^2^)” shows the corresponding r^2^ measure. Promoter and enhancer activity as well as eQTL (expression quantitative locus) activity is represented according to the examined brain tissues and hippocampus respectively. The HaploReg reports the regulatory information for the male and female fetal brain (FBF and FBM) as well as the following brain regions:

| CG: Cyngulate Gyrus |
| --- |
| AC: Anterior Caudate |
| DPC: Dorsolateral Prefrontal Cortex |
| GM: Germinal Matrix |
| SN: Substantia Nigra |
| AC: Anterior Caudate |
| ITL: Inferior Temporal Lobe |
| AG: Angular Gyrus |

| **“Anxiety only” versus “No Symptoms” Group** | | | | | | | | | | | | | | | | | | |
| --- | --- | --- | --- | --- | --- | --- | --- | --- | --- | --- | --- | --- | --- | --- | --- | --- | --- | --- |
| Gene | SNP | CHR | | BP(hg38) | | OR (95% CI) | | p-value | FDR q-value | Function | | SNP in LD | LD (r2) | Promoter histone marks in the brain | Promoter histone marks in hippocampus | Enhancer histone marks in the brain | Enhancer histone marks in hippocampus | eQTL correlated gene |
| GABRR1 | rs9342185 | 6 | | 89204419 | | 0.44  (0.25 - 0.78) | | 0.005 | 0.215 | intronic | | rs9342185 | 1 | NO | NO | NO | NO | NO |
| GABRR1 | rs4707529 | 6 | | 89208843 | | 0.47  (0.26 - 0.86) | | 0.015 | 0.241 | intronic | | rs4707529 | 1 | NO | NO | NO | NO | GABRR1 |
| GABRR1 | rs4707529 | 6 | | 89208843 | | 0.47  (0.26 - 0.86) | | 0.015 | 0.241 | intronic | | rs7739837 | 0.95 | NO | NO | NO | NO | GABRR1 |
| GABRR1 | rs4707529 | 6 | | 89208843 | | 0.47  (0.26 - 0.86) | | 0.015 | 0.241 | intronic | | rs7758893 | 0.82 | NO | NO | NO | NO | GABRR1 |
| GABRR1 | rs4707529 | 6 | | 89208843 | | 0.47  (0.26 - 0.86) | | 0.015 | 0.241 | intronic | | rs7773203 | 0.83 | NO | NO | NO | NO | GABRR1 |
| GABRR1 | rs4707529 | 6 | | 89208843 | | 0.47  (0.26 - 0.86) | | 0.015 | 0.241 | intronic | | rs282113 | 0.92 | NO | NO | CG | NO | GABRR1 |
| GABRR1 | rs4707529 | 6 | | 89208843 | | 0.47  (0.26 - 0.86) | | 0.015 | 0.241 | intronic | | rs43993 | 0.92 | NO | NO | CG | NO | GABRR1 |
| NOTCH1 | rs11145770 | 9 | | 136532614 | | 1.79  (1.10 - 2.89) | | 0.018 | 0.241 | intronic | | rs11145770 | 1 | AC, CG, DPC, GM | YES | all examined regions, FBF, FBM | YES | RP11-611D20.2; INPP5E |
| GABRR1 | rs7758893 | 6 | | 89206922 | | 0.53  (0.30 - 0.92) | | 0.025 | 0.241 | intronic | | rs7758893 | 1 | NO | NO | NO | NO | GABRR1 |
| GABRR1 | rs7758893 | 6 | | 89206922 | | 0.53  (0.30 - 0.92) | | 0.025 | 0.241 | intronic | | rs7739837 | 0.83 | NO | NO | NO | NO | GABRR1 |
| GABRR1 | rs7758893 | 6 | | 89206922 | | 0.53  (0.30 - 0.92) | | 0.025 | 0.241 | intronic | | rs4707529 | 0.82 | NO | NO | NO | NO | GABRR1 |
| GABRR1 | rs453503 | 6 | | 89190880 | | 0.52  (0.29 - 0.93) | | 0.028 | 0.241 | intronic | | rs453503 | 1 | AC | NO | NO | NO | ENSG00000146281.5; PM20D2 |
| GABRR1 | rs453503 | 6 | | 89190880 | | 0.52  (0.29 - 0.93) | | 0.028 | 0.241 | intronic | | rs7755627 | 0.98 | NO | NO | NO | NO | ENSG00000146281.5; PM20D2 |
| GABRR1 | rs453503 | 6 | | 89190880 | | 0.52  (0.29 - 0.93) | | 0.028 | 0.241 | intronic | | rs423463 | 1 | NO | NO | NO | NO | ENSG00000146281.5; PM20D2 |
| **“Comorbid symptoms” versus “No Symptoms” Group** | | | | | | | | | | | | | | | | | | |
| Gene | SNP | | CHR | | BP | | OR (95% CI) | p-value | FDR q-value | Function | SNP in LD | | LD (r2) | Promoter histone marks in the brain | Promoter histone marks in hippocampus | Enhancer histone marks in the brain | Enhancer histone marks in hippocampus | eQTL correlated gene |
| NOTCH1 | rs11145770 | | 9 | | 136532614 | | 2.21  (1.35 - 3.61) | 0.002 | 0.043 | intronic | rs11145770 | | 1 | AC, CG, DPC, GM | YES | all examined regions, FBF, FBM | YES | RP11-611D20.2; INPP5E |
| NOTCH1 | rs3013302 | | 9 | | 136537422 | | 2.15  (1.32 - 3.49) | 0.002 | 0.043 | intronic | rs3013302 | | 1 | SN, AC, CG, ITL, AG, DPC | YES | SN, AC, CG, ITL, AG, DPC, GM, FBM | YES | LOC286254; RP11-611D20.2 |
| NOTCH1 | rs3013302 | | 9 | | 136537422 | | 2.15  (1.32 - 3.49) | 0.002 | 0.043 | intronic | rs3013300 | | 0.87 | SN, AC, CG, ITL, AG, DPC | YES | SN, AC, CG, ITL, AG, DPC, GM | YES | RP11-611D20.2 |
| NOTCH1 | rs3013302 | | 9 | | 136537422 | | 2.15  (1.32 - 3.49) | 0.002 | 0.043 | intronic | rs3013301 | | 0.88 | SN, AC, CG, ITL, AG, DPC | YES | SN, AC, CG, ITL, AG, DPC, GM, FBM | YES | RP11-611D20.2 |
| NOTCH1 | rs3013302 | | 9 | | 136537422 | | 2.15  (1.32 - 3.49) | 0.002 | 0.043 | intronic | rs3013304 | | 0.98 | SN, AC, CG, ITL, AG, DPC | YES | SN, AC, CG, ITL, AG, DPC, GM, FBM, FBF | YES | RP11-611D20.2 |
| NOTCH1 | rs3013302 | | 9 | | 136537422 | | 2.15  (1.32 - 3.49) | 0.002 | 0.043 | intronic | rs3013305 | | 0.98 | SN, AC, CG, ITL, AG, DPC | YES | SN, AC, CG, ITL, AG, DPC, GM, FBM, FBF | YES | RP11-611D20.2 |
| NOTCH1 | rs13301342 | | 9 | | 136499893 | | 0.36  (0.16 - 0.77) | 0.009 | 0.097 | intronic | rs13301342 | | 1 | ST, AC, CG, ITL, NG | NO | SN, AC, CG, ITL, DPC, AG, FBM, FBF | YES | CARD9; INPP5E |
| NOTCH1 | rs13301342 | | 9 | | 136499893 | | 0.36  (0.16 - 0.77) | 0.009 | 0.097 | intronic | rs9632944 | | 0.94 | ST, AC, CG, ITL, AG | NO | SN, AC, CG, ITL, DPC, FBM, FBF | YES | INPP5E |
| NOTCH1 | rs13301342 | | 9 | | 136499893 | | 0.36  (0.16 - 0.77) | 0.009 | 0.097 | intronic | rs11574906 | | 0.99 | NO | NO | SN, AC, CG, ITL, AG, DPC | YES | PMPCA; CARD9; INPP5E |
| NOTCH1 | rs13301342 | | 9 | | 136499893 | | 0.36  (0.16 - 0.77) | 0.009 | 0.097 | intronic | rs12344155 | | 1 | NO | NO | SN, AC, CG, ITL, AG, DPC | YES | CARD9; INPP5E |
| NOTCH1 | rs13301342 | | 9 | | 136499893 | | 0.36  (0.16 - 0.77) | 0.009 | 0.097 | intronic | rs34383263 | | 0.97 | SN, CG, ITL | NO | SN, AC, CG, ITL, AG, DPC | YES | CARD9; INPP5E |
| NOTCH1 | rs13301342 | | 9 | | 136499893 | | 0.36  (0.16 - 0.77) | 0.009 | 0.097 | intronic | rs13300218 | | 0.99 | ITL | NO | SN, AC, CG, ITL, AG, DPC | YES | PMPCA; CARD9; INPP5E |
| NOTCH1 | rs13301342 | | 9 | | 136499893 | | 0.36  (0.16 - 0.77) | 0.009 | 0.097 | intronic | rs7870145 | | 0.83 | ITL | NO | SN, AC, CG, ITL, AG, DPC | YES | SNAPC4 |
| NOTCH1 | rs13301342 | | 9 | | 136499893 | | 0.36 (0.16 - 0.77) | 0.009 | 0.097 | intronic | rs11574902 | | 0.96 | AC, ITL | NO | SN, AC, CG, ITL, AG, DPC | YES | PMPCA; CARD9; INPP5E |
| NOTCH1 | rs13301342 | | 9 | | 136499893 | | 0.36  (0.16 - 0.77) | 0.009 | 0.097 | intronic | rs11145765 | | 0.93 | AC, ITL, AG | NO | SN, AC, CG, ITL, AG, DPC | YES | PMPCA; CARD9; INPP5E |
| NOTCH1 | rs13290979 | | 9 | | 136531182 | | 1.92  (1.18 - 3.15) | 0.009 | 0.097 | intronic | rs13290979 | | 1 | all examined regions, FBF, FBM | YES | all examined regions, FBF, FBM | YES | RP11-611D20.2 |
| NOTCH1 | rs13290979 | | 9 | | 136531182 | | 1.92  (1.18 - 3.15) | 0.009 | 0.097 | intronic | rs13290840 | | 0.98 | all examined regions, FBF, FBM | YES | all examined regions, FBF, FBM | YES | RP11-611D20.2 |
