# Supplementary figures and images for: Implication of NOTCH1 gene in susceptibility to anxiety and depression among sexual abuse victims
Source: Transl Psychiatry. 2016 Dec 13;6(12):e977–. doi: 10.1038/tp.2016.248 (PMC5290341; doi:10.1038/tp.2016.248)

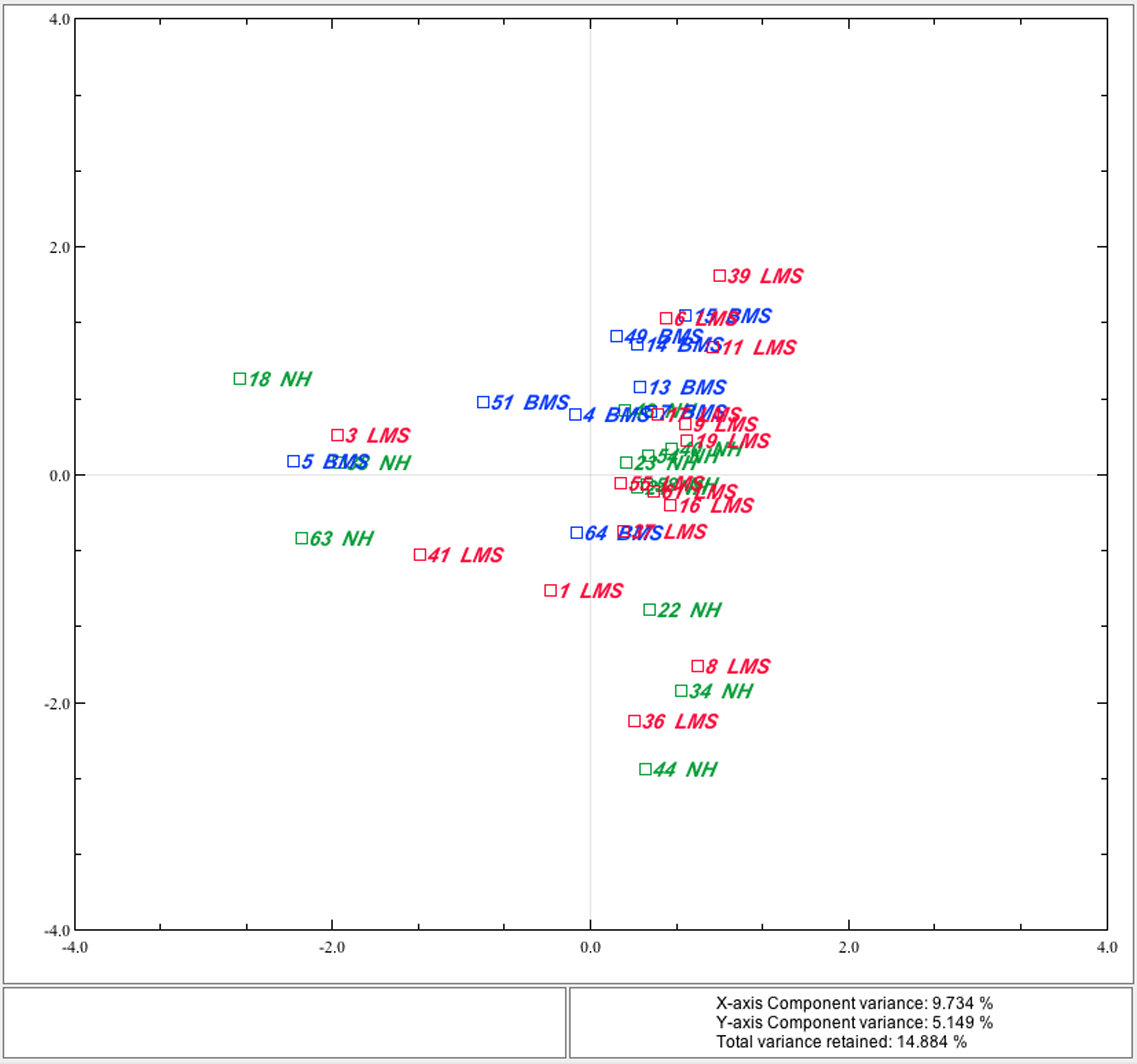

Supplement: Supplementary Figure 1 [file tp2016248x4.tif]

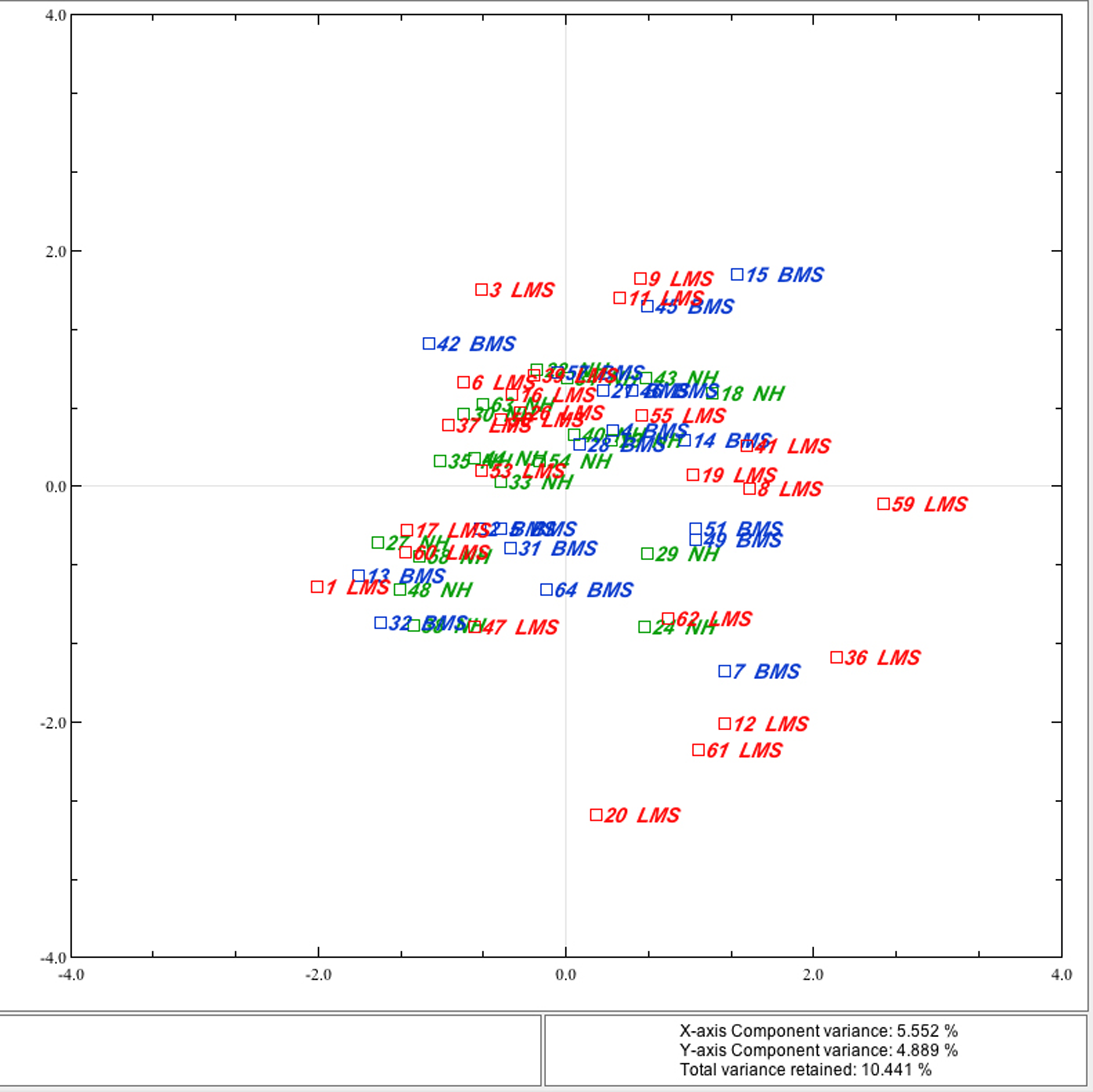

Supplement: Supplementary Figure 2 [file tp2016248x5.tif]
