# Supplementary material for: Implication of NOTCH1 gene in susceptibility to anxiety and depression among sexual abuse victims
Source: Transl Psychiatry. 2016 Dec 13;6(12):e977–. doi: 10.1038/tp.2016.248 (PMC5290341; doi:10.1038/tp.2016.248)
Supplement: Supplementary Figure 3 [file tp2016248x6.docx]

**Representative population sample**

N= **706**

Fulfilled sexual abuse criteria

n= **97**

Successfully genotyped

n= **92**

Provided saliva sample

n = **306**

**Sexually abused sample from**

**support centers**

N= **537**

Successfully genotyped

n= **269**

**Total study sample**

(Sexually abused + successfully genotyped)

n= **361**

Provided saliva sample

n= **306**
